# Unraveling Atypical Osteomyelitis: A Rare Group B Streptococcus Infection in an Adolescent With Diagnostic Dilemma

**DOI:** 10.7759/cureus.100069

**Published:** 2025-12-25

**Authors:** Mudassir A Khan, Matthew Schade, Lauren Tufts, Troy Wallace, Mariana M Lanata

**Affiliations:** 1 Pediatrics, Marshall University Joan C. Edwards School of Medicine, Huntington, USA; 2 Medicine-Pediatrics, Marshall University Joan C. Edwards School of Medicine, Huntington, USA; 3 Pediatric Infectious Diseases, Marshall University Joan C. Edwards School of Medicine, Huntington, USA

**Keywords:** adolescent, bacteremia, differential diagnosis, osteomyelitis, sacroiliac joint, streptococcus agalactiae, urinary incontinence

## Abstract

Group B Streptococcus (GBS) is a well-recognized pathogen in neonates and older adults but is rare in adolescents, particularly as a cause of musculoskeletal infection. We describe the case of a previously healthy 16-year-old female patient with GBS sacroiliac septic arthritis and osteomyelitis presenting with progressive hip and back pain complicated by acute urinary incontinence. Initial evaluation was confounded by nonspecific symptoms, under-recognized inflammatory markers, and incidental spinal imaging findings, resulting in diagnostic delay. Blood cultures ultimately identified GBS, and targeted antimicrobial therapy led to clinical improvement. Subsequent imaging and laboratory findings raised concern for chronic osteomyelitis, prompting prolonged therapy with full recovery. This case highlights an unusual presentation of adolescent GBS osteomyelitis involving the sacroiliac joint and acute urinary incontinence, an association not previously reported. It underscores the importance of maintaining a broad differential, interpreting laboratory abnormalities in context, avoiding diagnostic anchoring, and obtaining site-directed imaging in adolescents with persistent musculoskeletal pain and systemic inflammation.

## Introduction

Osteomyelitis has an estimated incidence of 1.2-1.3 per 100,000 individuals, affecting both pediatric and adult populations [1,2]. According to the American Academy of Pediatrics, the annual incidence of acute osteomyelitis in children rose from 1.0 to 1.8 per 100,000 in 2007-2008 and 2015-2016. The highest incidence was reported in children aged 10-14 years (33.2%), while adolescents aged 15-18 years accounted for 13.3% of cases [3].

*Staphylococcus aureus* remains the predominant causative pathogen in hematogenous osteomyelitis, with methicillin-sensitive* S. aureus* (MSSA) accounting for approximately 61.7% of cases and methicillin-resistant *S. aureus *(MRSA) representing about 38.3%, followed by *Streptococcus pyogenes* and *Streptococcus pneumoniae* [1,2,4]. However, in neonates and older adults, *Streptococcus agalactiae* (Group B Streptococcus, GBS) is also a significant pathogen associated with osteomyelitis [1,2,5].

According to the Centers for Disease Control and Prevention (CDC), approximately 28,000 cases of invasive GBS disease occur annually in the United States, making it one of the key pathogens under national surveillance. The 2022 CDC report estimated an overall incidence of 8.6 cases per 100,000 population, with the highest burden seen in infants and older adults. Conversely, adolescents accounted for the lowest incidence, at just 0.2%, highlighting the rarity of GBS infection in this age group [6].

In this case, we present a previously healthy 16-year-old female child who developed GBS-induced sacroiliac osteomyelitis, a rare diagnosis in adolescents. The development of urinary incontinence, an uncommon manifestation of osteomyelitis, along with nonspecific early symptoms and incidental spinal imaging findings, contributed to diagnostic difficulty. This case highlights the challenges of recognizing uncommon pathogens in the setting of atypical clinical presentations.

## Case presentation

A previously healthy 16-year-old female patient initially presented with lower extremity musculoskeletal pain. The discomfort first became noticeable two weeks earlier, following her return from cheer practice, and was presumed to be of muscular origin secondary to overuse. Despite rest and over-the-counter analgesics, her symptoms persisted, prompting an orthopedic evaluation. Examination revealed lateral hip tenderness and pain with hip movement, while hip and knee radiographs obtained at that visit were unremarkable. She was reassured and advised to continue analgesics and physical therapy. An outpatient follow-up was scheduled within a week, with plans to obtain hip and knee MRI imaging if no clinical improvement was noted.

The next day, she presented to the emergency department (ED) with worsening left hip pain and new-onset difficulty bearing weight. Laboratory evaluation revealed leukocytosis (white blood cell (WBC) 14.28 ×10³/µL) with neutrophilic predominance, along with elevated inflammatory markers, including a C-reactive protein (CRP) of 4.1 mg/L and erythrocyte sedimentation rate (ESR) of 49 mm/hr. Hip and lumbar spine radiographs obtained were unremarkable. Her pain and weight-bearing ability improved significantly after analgesic therapy given in the ED. The Orthopedics team evaluated her in the ED and provided reassurance. Following shared decision-making with the patient and her family, she was discharged with instructions to continue outpatient follow-up and physical therapy.

**Figure 1:**
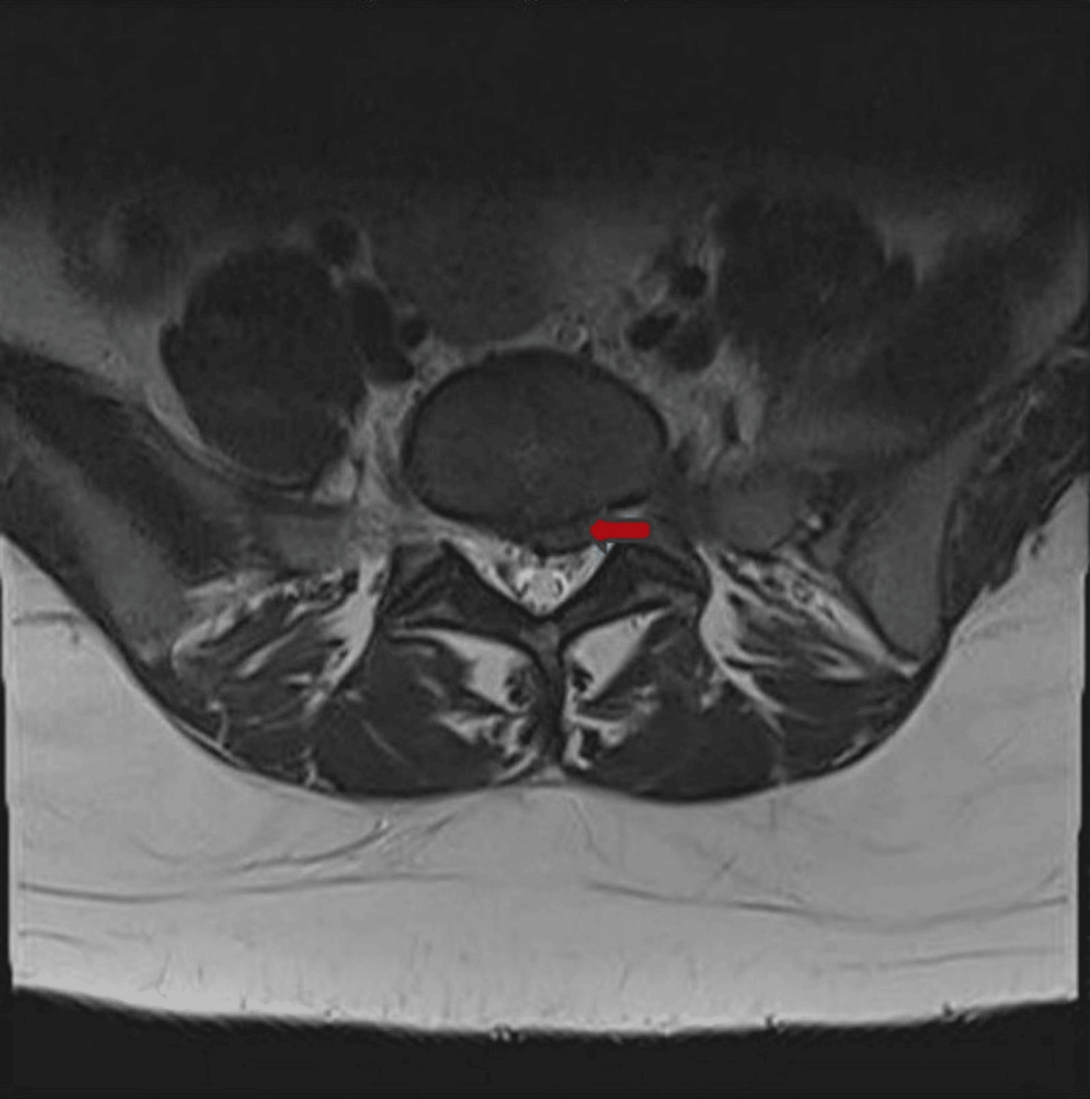
MRI of lumbar spine on admission showing left paracentral disc herniation.

Within 48 hours, she returned to the ED with fever (peaking at 102°F), lower back swelling, and recurrence of severe left hip and knee pain with difficulty bearing weight. She also experienced two episodes of bladder incontinence, which heightened concern and prompted reevaluation. A detailed neurologic examination revealed no additional deficits. Repeat laboratory testing demonstrated worsening inflammation, with WBC 17.8 ×10³/µL, CRP 21.4 mg/L, and ESR 100 mm/hour, while her metabolic panel and urinalysis remained unremarkable. An MRI of the lumbar spine showed an L5-S1 disc herniation, mild L4-L5 spinal stenosis, and displacement of the left S1 nerve root (Figure 1).

Given her clinical deterioration and evolving findings, she was admitted to the inpatient pediatric unit for further evaluation and comprehensive management. A detailed history obtained by the inpatient team further revealed recent unprotected sexual contact with one partner, although she reported no genitourinary symptoms aside from urinary incontinence. Neurosurgery was consulted due to the urinary incontinence and radiologic evidence of disc herniation. A broad infectious workup was initiated, including blood and urine cultures, a urine pregnancy test, sexually transmitted infection screening (*Neisseria gonorrhoeae*, *Chlamydia*, HIV, hepatitis panel, and syphilis), a viral panel, and testing for Lyme disease and Epstein-Barr virus (EBV).

Blood cultures collected in the ED grew GBS within 10 hours, while the remainder of the infectious workup was negative. The Infectious Diseases team was consulted and recommended initiating intravenous ampicillin, obtaining daily blood cultures, and performing additional imaging to identify the source of bacteremia. Given the predominance of hip pain, an MRI of the hip and spine was advised to evaluate for osteomyelitis or a paraspinal abscess. A repeat blood culture again grew GBS within 14 hours. Neurosurgery deferred immediate surgical intervention for the disc herniation in light of the ongoing infection, with plans to intervene only if urinary incontinence did not respond to medical management.

**Figure 2:**
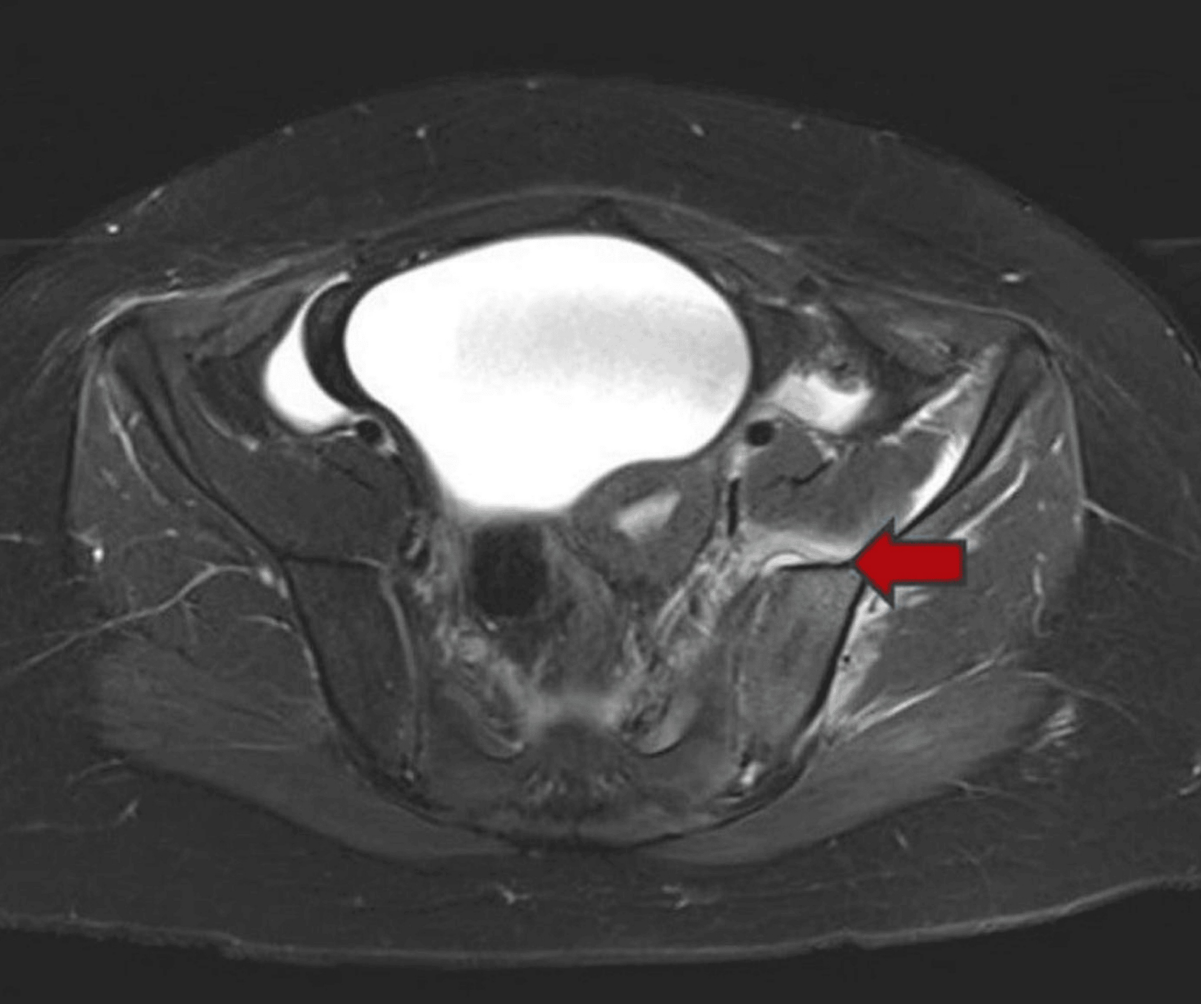
Pelvic MRI (day 3) showing left sacroiliac septic arthritis with adjacent osteomyelitis and abscess

Over the next 48 hours, the patient showed significant clinical improvement. Her fevers subsided, pain decreased steadily, she began ambulating with assistance, and she gradually regained bladder continence. Laboratory markers trended positively, with declining WBC, CRP, and ESR levels, while repeat blood cultures remained sterile. MRI of the pelvis with contrast revealed left sacroiliac septic arthritis, adjacent osteomyelitis, myositis, and an 8 x 4 mm abscess (Figure 2).

Interventional Radiology performed a diagnostic CT-guided abscess drainage, and cultures confirmed GBS infection. Intravenous ampicillin was transitioned to oral amoxicillin after six days of therapy. Plans were made for discharge with a four-week course of oral amoxicillin from the first negative blood culture following source control, consistent with clinical guidelines.

**Figure 3:**
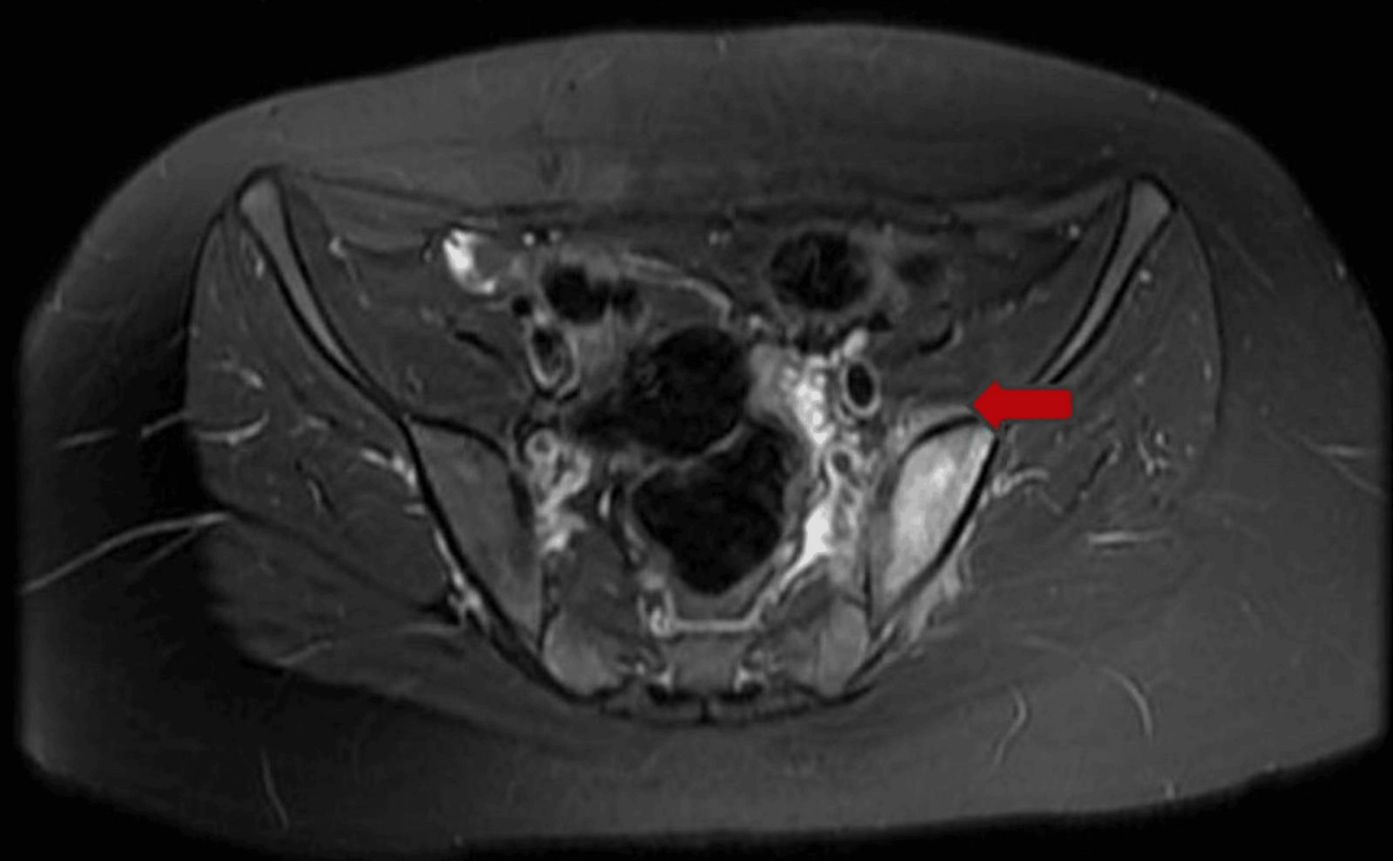
Follow-up hip MRI (day 72) with residual left sacral and iliac marrow edema

Upon discharge, the patient maintained close follow-up with her primary care physician and specialists, including Neurosurgery, Infectious Diseases, and Orthopedics. She adhered to the prescribed antibiotic regimen, completing the full four-week course with normalization of laboratory markers prior to discontinuation. She also participated in structured outpatient physical therapy. A repeat MRI of the hip demonstrated significant improvement in myositis, although persistent edema remained in the left sacral ala and ilium (Figure 3). 

Within a few days after discontinuation of antibiotics, the patient developed fever, sore throat, and body aches. Based on her clinical symptoms, her primary care physician diagnosed her with streptococcal pharyngitis and placed her on amoxicillin without confirmatory testing. Her mother informed the Infectious Diseases team, prompting re-evaluation. While her CBC remained normal, her ESR was elevated at 48 mm/hour, with minimal CRP elevation at 1.3 mg/L. She still complained of mild muscle pain but had no further urinary incontinence. The disproportionately elevated ESR compared to CRP raised concern for chronic osteomyelitis, with an additional eight-week course of amoxicillin prescription.

**Figure 4:**
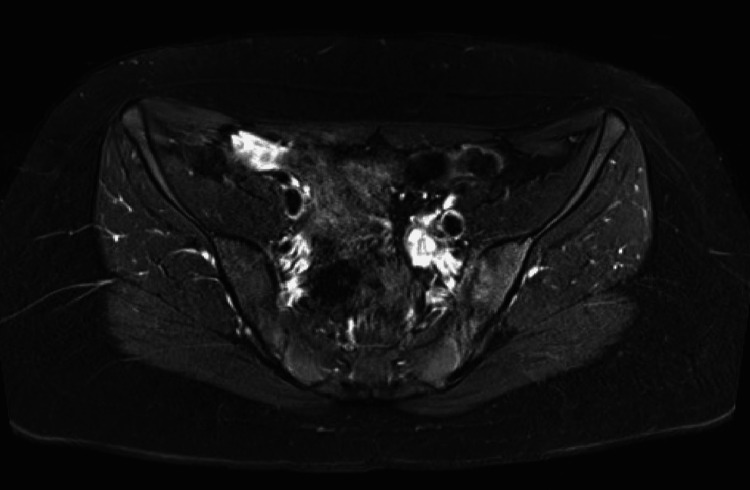
Post-treatment pelvic MRI demonstrating resolution of left sacroiliac infection with mild residual joint enhancement

The patient sought a second opinion at a tertiary children’s hospital, where she was evaluated by Pediatric Infectious Disease and Neurosurgery specialists. After a comprehensive assessment, the consulting teams confirmed the diagnosis of chronic osteomyelitis and endorsed the ongoing treatment plan. She continued the second course of amoxicillin, with serial laboratory monitoring demonstrating normalization of inflammatory markers and resolution of pain. A follow-up pelvic MRI obtained after completion of the prolonged antimicrobial therapy confirmed complete resolution of the sacroiliac septic arthritis and adjacent osteomyelitis (Figure 4).

The temporal variations in white blood cell count and inflammatory parameters are outlined in Table 1, illustrating the patient’s inflammatory trajectory across the disease course.

**Table 1:** Summary of WBC, ESR, CRP, and blood culture results during entire course of disease process. WBC: white blood cells; ESR: erythrocyte sedimentation rate; CRP: C-reactive protein

Date	WBC Value (x10^9^/L); Reference Range: 4–11	ESR Value (mm/hour); Reference Range: 0–20	CRP Value (mg/L); Reference Range: <0.5	Blood Cultures
2 days before admission	14.28	49	4.10	-
Day of admission	16.71	100	19.80	Gram Stain: Gram-positive cocci in chains, positive in 10 and again in 14 hours. Culture: Group B Streptococcus
Day 2	14.04	107	18.00	-
Day 3	10.87	-	15.40	No growth at 5 days.
Day 4	9.13	-	11.50	No growth at 5 days.
Day 6 (Day of discharge)	9.72	98	4.30	-
Day 16	10.27	57	0.50	-
Day 31	7.20	17	0.13	-
Day 72 (Re-Initiation of antibiotics)	8.86	48	1.30	-
Day 82	8.01	26	0.10	-
Day 103	9.30	23	<0.5	-

## Discussion

Adolescent-onset GBS disease is exceptionally rare, with most cases occurring in neonates and older adults. Literature on GBS infections beyond the neonatal period is limited, particularly in pediatrics and adolescents. Edwards et al. reported only one case of calcaneal osteomyelitis in a healthy 11-year-old within a 10-patient series [7]. Another case involved a 16-year-old African American male with right knee osteomyelitis, associated with a benign non-ossifying bone fibroma. Histopathology further suggested possible underlying Langerhans cell histiocytosis, adding complexity to the presentation [8].

Urinary complications in pediatric GBS musculoskeletal infections have not been previously reported. However, upon review of adult literature, Bauer et al. described a case of pyogenic vertebral osteomyelitis caused by GBS in a 54-year-old patient, which was attributed to a complication of an existing urinary tract infection [9]. Similarly, McKenna and O’Brien reported a case of GBS bacteremia with sacroiliitis in a 37-year-old multigravida woman following complications of a second-trimester dilation and evacuation [10]. Both cases presented with musculoskeletal pain, fever, and bacteremia, mirroring our patient’s symptoms. However, neither case involved urinary incontinence, which further emphasizes the uniqueness of our case, given the patient’s age, the atypical infection site, and the development of acute urinary incontinence as a complication.

This case reflects the challenge of distinguishing early infection from routine, nonspecific musculoskeletal symptoms. The patient's persistent knee and hip pain were initially attributed to musculoskeletal strain due to high activity levels. Although leukocytosis and elevated inflammatory markers were present, the nonspecific nature of her early presentation made an infectious etiology less apparent to clinicians. Upon admission, despite the presence of fever and abnormal laboratory results, clinical attention shifted to urinary incontinence, prompting targeted lumbar spine MRI imaging. This diagnostic anchoring limited further evaluation of her primary complaint of hip pain, ultimately contributing to a delay in identifying the underlying osteomyelitis and abscess.

The mild lumbar disc herniation observed on MRI further misdirected clinical focus, prompting neurosurgical consultation and potential surgical intervention. While disc herniation can contribute to incontinence, studies show such findings are often incidental. Brinjikji et al. reported disc protrusions in 29% of asymptomatic 20-year-olds, increasing to 43% by age 80 [11]. Kanayama et al. found herniation rates from 0.5% at L1-2 to 35% at L5-S1 [12]. The herniation was unlikely to be acute, and her urinary incontinence, hip pain, and fever were more indicative of an infectious etiology. Furthermore, her laboratory findings, including leukocytosis and markedly elevated inflammatory markers, further supported this concern.

The detection of GBS bacteremia prompted an infectious diseases consultation, leading to hip imaging that confirmed osteomyelitis. While osteomyelitis in children typically affects long bones such as the femur and tibia, involvement of the sacroiliac joint is atypical and may be less readily recognized [1]. Importantly, not all osteomyelitis cases present with bacteremia, with a reported incidence of approximately 31.2% based on systematic review data [2]. In this case, the presence of GBS bacteremia facilitated earlier recognition of infection, allowing for timely targeted therapy and intervention. Without bacteremia, the continued emphasis on spinal pathology and lack of early hip imaging could have further delayed diagnosis, potentially increasing the risk of complications.

Whether this patient had chronic osteomyelitis remains debatable. The recurrence of her symptoms may have been attributable to acute streptococcal pharyngitis, as they resolved rapidly with initiation of amoxicillin; however, this antimicrobial therapy also provided coverage for potential persistent GBS osteomyelitis. The disproportionately elevated ESR compared to her CRP supported a more chronic inflammatory process rather than an acute infection, in which the opposite pattern would typically be expected. In conjunction with residual imaging abnormalities, these findings were sufficiently concerning to justify treatment as chronic osteomyelitis, while acknowledging that radiographic changes often lag behind clinical improvement. Ultimately, the patient made a full recovery and had full resolution of findings in repeat imaging.

## Conclusions

This case underscores the importance of maintaining clinical vigilance-resisting diagnostic anchoring, embracing a truly comprehensive, whole-patient perspective, and continually revisiting the working diagnosis, particularly when confronted with atypical or evolving presentations.
